# Abaloparatide Has the Same Catabolic Effects on Bones of Mice When Infused as PTH (1–34)

**DOI:** 10.1002/jbm4.10710

**Published:** 2023-01-05

**Authors:** Carole Le Henaff, Brandon Finnie, Maria Pacheco, Zhiming He, Joshua Johnson, Nicola C. Partridge

**Affiliations:** ^1^ Department of Molecular Pathobiology New York University College of Dentistry New York New York USA

**Keywords:** ABALOPARATIDE, BONE CATABOLISM, HIGH BONE TURNOVER, PTH

## Abstract

Abaloparatide is a peptide analog of parathyroid hormone‐related protein (PTHrP 1–34) and was approved in 2017 as the second osteoanabolic peptide for treating osteoporosis. We previously showed that intermittent abaloparatide is equally as effective as PTH (1–34). This study was designed to compare the catabolic effects of PTH (1–34) and abaloparatide on bone in young female wild‐type mice. Two‐month‐old C57Bl/6J female mice were continuously infused with human PTH (1–34) or abaloparatide at 80 μg/kg BW/day or vehicle for 2 weeks. At euthanasia, DEXA‐PIXImus was performed to assess bone mineral density (BMD) in the whole body, femurs, tibiae, and vertebrae. Bone turnover marker levels were measured in sera, femurs were harvested for micro–computer tomography (μCT) analyses and histomorphometry, and tibiae were separated into cortical and trabecular fractions for gene expression analyses. Our results demonstrated that the infusion of abaloparatide resulted in a similar decrease in BMD as infused PTH (1–34) at all sites. μCT and histomorphometry analyses showed similar decreases in cortical bone thickness and BMD associated with an increase in bone turnover from the increased bone formation rate found by in vivo double labeling and serum P1NP and increased bone resorption as shown by osteoclast numbers and serum cross‐linked C‐telopeptide. Trabecular bone did not show major changes with either treatment. Osteoblastic gene expression analyses of trabecular and cortical bone revealed that infusion of PTH (1–34) or abaloparatide led to similar and different actions in genes of osteoblast differentiation and activity. As with intermittent and in vitro treatment, both infused PTH (1–34) and abaloparatide similarly regulated downstream genes of the PTHR1/SIK/HDAC4 pathway such as *Sost* and *Mmp13* but differed for those of the PTHR1/SIK/CRTC pathway. Taken together, at the same dose, infused abaloparatide causes the same high bone turnover as infused PTH (1–34) with a net resorption in female wild‐type mice. © 2023 The Authors. *JBMR Plus* published by Wiley Periodicals LLC on behalf of American Society for Bone and Mineral Research.

## Introduction

Osteoporosis is a highly prevalent disease characterized by decreased bone mass with altered bone microarchitecture associated with an increase in the risk of fractures.^(^ ^1^ ^)^ It results from an imbalance between bone formation and bone resorption with a decrease in bone formation by osteoblasts and an increase in bone resorption by osteoclasts due, in most cases, to a loss of hormone, such as estrogen or testosterone, and/or aging. The most commonly used treatment for osteoporosis are bisphosphonates,^(^ ^2^ ^)^ but they only prevent bone resorption. One important challenge in osteoporosis is to improve bone formation by rebuilding missing bone.

Teriparatide, or the first 34 amino acids of PTH, was the first US Food and Drug Administration (FDA)‐approved osteoanabolic therapy,^(^ ^3^ ^)^ and it continues to be investigated.^(^ ^3^, ^4^ ^)^ During normal homeostatic conditions, the balance between bone formation and bone resorption is equal.^(^ ^5^ ^)^ PTH (1–34), when administered either continuously or intermittently, alters this balance. With intermittent PTH (1–34), the balance is positive with a greater increase in bone formation than bone resorption. This results in improved bone mass and microarchitecture.^(^ ^6^, ^7^ ^)^ Nevertheless, patients show greater osteoanabolic effects of PTH (1–34) in the first 1–2 years of treatment, which is then taken over by an increase in bone resorption, creating what some have called “the anabolic window.”^(^ ^7^, ^8^, ^9^, ^10^ ^)^ On the other hand, the balance is negative with continuous infusion of PTH (1–34) or elevated PTH levels in hyperparathyroidism. Bone turnover is high and bone resorption overwhelms bone formation. When used in rodents, this model provides a better understanding of PTH's catabolic effects.^(^ ^11^ ^)^ Continuous infusion of PTH in rodents mimics parathyroid bone diseases, such as hyperparathyroidism in humans.

Abaloparatide, an analog of parathyroid hormone‐related protein (PTHrP 1–34), was approved by the FDA in 2017 as a second osteoanabolic peptide treatment. Abaloparatide has 76% homology to PTHrP (1–34) and 41% homology to PTH (1–34). The peptide was created to have enhanced stability and potent bone anabolic activity and low calcium‐mobilization potential.^(^ ^12^, ^13^ ^)^ ACTIVE clinical trials reported that abaloparatide was similar, and perhaps superior, to teriparatide in increasing bone mineral density (BMD) in osteoporotic postmenopausal women, and these subjects also showed lower serum cross‐linked C‐telopeptide (CTX) levels, the marker for bone resorption, than patients treated with teriparatide.^(^ ^14^, ^15^ ^)^ Nevertheless, after 18 months, both need to be followed by an antiresorptive agent to avoid new bone loss after the discontinuation of anabolic treatments.^(^ ^13^, ^16^, ^17^ ^)^

PTH (1–34) and abaloparatide both bind the same G‐protein‐coupled receptor, PTH/PTH‐related peptide receptor type 1 (PTHR1). This receptor is highly expressed in several organs, such as bone, kidney, and cartilage, and in other tissues but at lower levels.^(^ ^18^, ^19^ ^)^ The G_αS_/cyclic adenosine monophosphate (cAMP)/protein kinase A (PKA) pathway is known to mediate the osteoanabolic response of PTH (1–34).^(^ ^11^ ^)^ More precisely, both are thought to bind similarly to the G‐protein‐coupled receptor form (RG). However, PTH (1–34) has a greater affinity for the non‐G‐protein‐coupled receptor R^0^ than abaloparatide, which is thought to cause PTH (1–34) to produce cumulatively greater signaling and biological responses than abaloparatide. This produces prolonged cyclic adenosine monophosphate (cAMP) levels, which is thought to cause relatively greater bone resorption.^(^ ^20^ ^)^ Even if both bind the same receptor, downstream PTHR1 effects are not the same with differences in cAMP levels, PKA activation, and, as a consequence, gene expression exerted by their anabolic effects.^(^ ^20^, ^21^ ^)^ Abaloparatide has been shown to be a weaker activator of cAMP/PKA/CREB signaling than PTH (1–34), associated with a lower stimulation of *c‐Fos* and *Rankl* expression. This was demonstrated in mouse osteoblasts in vitro and in vivo.^(^ ^21^ ^)^ The improved anabolic actions of abaloparatide are thought to be due to a lower effect on resorption observed in rat bones and in clinical studies.^(^ ^22^, ^23^, ^24^ ^)^ Nonetheless, there have been very few head‐to‐head studies of abaloparatide and PTH (1–34).^(^ ^14^, ^25^, ^26^ ^)^ Most studies have compared the effects of several concentrations of abaloparatide on bone in rats and in monkeys without comparing it with PTH (1–34).^(^ ^24^, ^27^, ^28^, ^29^ ^)^ Only two studies compared both peptides at the same dose in wild‐type animals.^(^ ^30^, ^31^ ^)^ Both show a similar anabolic effect for PTH (1–34) and abaloparatide in male and female wild‐type mice. In our previous study in wild‐type male mice, PTH (1–34) and abaloparatide by daily injection also caused a similar increase in osteoclast number in trabecular bone, where turnover is most important in anabolic treatments.^(^ ^30^ ^)^ The effects of PTH (1–34) and abaloparatide as osteoanabolic treatments are complex and need to be completely elucidated. In this work, we determined the catabolic effects of infused PTH (1–34) and abaloparatide at the same dosage, 80 μg/kg BW/day, in intact female mice to determine whether abaloparatide would have a less catabolic effect than PTH (1–34).

## Materials and Methods

### Animals

Two‐month‐old C57Bl/6J female mice were bred in our animal facility. We fed them with a mouse standard diet (PicoLab® Rodent Diet 20, 5053, LabDiet) containing calcium (0.81%), phosphorus (0.63%), and vitamin D (2.2 IU/g). The mice were housed on a 12‐h dark /light cycle with standard rodent chow and water ad libitum. All mouse‐related experimental procedures were performed in accordance with an approved protocol of the Institutional Animal Care and Use Committee of New York University Grossman School of Medicine. In each litter, mice were chosen randomly for PTH or abaloparatide or vehicle treatment. Mice (10 randomized mice per group) were infused with human PTH (1–34) at a standard dose (80 μg/kg BW/day, 86% peptide content, Bachem) or abaloparatide (80 μg/kg BW/day, 84.1% peptide content, Bachem) or the vehicle (acetic acid, 100 μM in PBS). Peptides or vehicle was delivered at a rate of 0.25 μl/h for 2 weeks by surgically implanted ALZET osmotic pump model‐1002 (DURECT Corp., Cupertino, CA, USA) subcutaneously in the midscapular region of mice following the manufacturer's instructions. To label bone, control and treated mice were given tetracycline (20 mg/kg; Sigma, St. Louis, MO, USA) and calcein (10 mg/kg; Sigma, St. Louis, MO, USA) by intraperitoneal injection, respectively, at days 4 and 1 before euthanasia with a lethal injection of ketamine/xylazine (7.5 mg/375 μg per animal).

### DEXA‐PIXImus analyses

At euthanasia, the mice were weighed and assessed by DEXA‐PIXImus (dual energy x‐ray absorptiometry) for BMD by an independent blinded person. On the day of BMD measurement, just prior to use, the machine is warmed up and the phantom is measured to standardize the machine. The program is composed of several impulses of two different X‐ray beams: LE ('Low Energy' X‐ray attenuation, voltage at 35 kV, current at 0.5 mA for 15 seconds) and HE ('High Energy' X‐ray attenuation, voltage at 80 kV, current at 0.5 mA for 3 seconds). Following warm‐up and standardization, the mouse is laid down with the head in the circular groove of the specimen tray. The tail is laid away from the main body to be sure that it does not cross any of the long bones. Then, with gentle traction, the tail is pulled to straighten the spine and ensure that the skull is parallel to the sagittal plane. Legs are moved away from the body with the front legs at a 45° angle to the spine to prevent overlap and femurs at a 90° angle to the spine. Knees are set to an angle of 90°. Every image is checked immediately after each DEXA scan. The mouse is rescanned if necessary due to poor‐quality images or movement. Accurate measurements were taken, always in the same fashion, by a person blinded to the animal. BMD was measured by defined region of interest of the whole body (excluding the skull and the ear tag), the right femurs (from the hip to the middle of the knee), the right tibiae (the middle of the knee to the middle of the ankle), and lumbar vertebrae (from the ribs to the hips). Absolute BMD values were expressed in g/cm^2^.

### Serum analyses

To collect blood samples by cardiac puncture, mice were injected with a lethal dose of ketamine/xylazine (7.5 mg/375 μg per animal). Death was confirmed by subsequent cervical dislocation. The blood was allowed to clot at room temperature, then centrifuged at 2040 *g* for 10 minutes to separate sera. The samples were frozen before ELISA analysis for the N‐terminal propeptide of type 1 procollagen (P1NP) levels (Immunodiagnostic Systems Inc., Boldon, UK) or the C‐terminal crosslinking telopeptide of type I collagen (CTX, Immunodiagnostic Systems Inc., Boldon, UK). These are established markers of bone formation and bone resorption, respectively. All analyses were done blinded.

### Micro‐computed tomography

After euthanasia, we dissected femurs, cleaned them of soft tissue, and fixed/stored them in 70% ethanol. The samples were scanned in batches of six at a nominal resolution (pixels) of 9.7 μm using a high‐resolution micro‐computer tomography μCT system (μCT, SkyScan 1172, SkyScan, Ltd., Kartuizersweg, Kontich, Belgium). The following imaging parameters were used: 60 kV, 167 μA, and an aluminum 0.5‐mm filter. All images were reconstructed using NRecon (SkyScan, Kartuizersweg, Kontich, Belgium). This is a three‐dimensional (3D) morphometry evaluation program, and we used specific parameters such as beam‐hardening correction of 40, ring artifact correction of 7, and Gaussian smoothing (factor 1). The reconstructed data were binarized using a thresholding of 80–255 using CTAn software (SkyScan, Kartuizersweg, Kontich, Belgium). This allowed us to analyze cortical bone by two‐dimensional analyses. For 3D volumetric analyses of trabecular bone, reconstructed images were binarized using a threshold of 60–255. The BMD of trabecular and cortical bone was determined from the binary data based on a calibration curve of calcium hydroxyapatite standards which were measured at the same time and with the same parameters used for the experimental samples. The μCT measurements follow the guidelines reported by Bouxsein et al.^(^ ^32^ ^)^ For cortical bone architecture, we examined a 600‐μm volume corresponding to 62 slices of the mid‐diaphysis that began immediately distal to the third trochanter. For trabecular bone microarchitecture, a 1940‐μm volume corresponding to 200 slices of the mid‐metaphysis were chosen. This began 20 slices below the growth plate. All analyses were done blinded by two different persons.

### Histomorphometry analyses

For histomorphometry analyses, the same fixed femurs were used after uCT scanning. Femurs were fixed in 70% ethanol, dehydrated, and embedded in methyl methacrylate (Polysciences, Warrington, PA, USA). We used a Leica RM2265 microtome to cut longitudinal tissue sections (5 and 10 μm thick). Histomorphometry analysis of femurs was performed following established protocols.^(^ ^33^, ^34^, ^35^ ^)^ Sections 5 μm thick were stained with Masson's trichrome to analyze structural parameters such as bone volume/tissue volume (BV/TV), trabecular number (Tb.N), thickness (Tb.Th), separation (Tb.Sp), and osteoid volume (OV/BV). Tartrate‐resistant acid phosphatase (TRACP) staining was done to detect active osteoclasts. Unstained 10‐μm sections were used to assess dynamic parameters from the double fluorochrome injections (calcein and tetracycline), such as mineral apposition rate (MAR, distance between two labels), double‐labeled surface (mineralizing surface or MS, single and double labeling/2 per bone surface, BS), and bone formation rate (BFR, MAR*MS/BS). All quantitative analyses were performed with the BioQuant image analysis system (Nashville, TN, USA). All histomorphometry measurements were calculated and conducted following the standard nomenclature approved by the American Society for Bone and Mineral Research.^(^ ^34^, ^35^ ^)^ All analyses were done blinded by two different persons.

### RNA isolation and reverse transcriptase quantitative polymerase chain reaction (RT‐qPCR) analysis

Tibiae were dissected and soft tissues removed. Next, we cut off the distal and proximal ends of the tibiae. These correspond to subcortical trabecular rich regions and the growth plates. Following this, we centrifuged the bone and collected the bone marrow in a new Eppendorf tube. After completely removing the bone marrow, the remaining cortical bone was selected as being osteocyte‐rich. Total RNA was extracted from the trabecular‐rich and cortical fractions using a TRIzol kit (Sigma, Boldon, UK) followed by cDNA synthesis (from 1 μg of total RNA) using TaqMan® reverse transcription reagents (Life Technologies, Inc., Carlsbad, CA, USA). We used SYBR® Green Master Mix for reverse transcriptase quantitative polymerase chain reaction (RT‐qPCR) and a Mastercycler® realplex^2^ instrument (Eppendorf, Hamburg, Germany). We calculated mRNA expression using the standard fold change formula 2^−(ΔΔCt)^. The levels of mRNA expression were normalized to geometrical means with *Gapdh* and *Hprt* expression (Table 1) as internal controls (2^−(ΔCt)^) and then expressed as fold values compared with the vehicle‐treated mice (2^−(ΔΔCt)^). The RT‐qPCR primers are listed in Table 1.

**Table 1:** Mouse Primers

	Forward	Reverse
Gapdh	GACTGTGGATGGCCCCTCTG	CGCCTGCTTCACCACCTTCT
Hprt	GGAGCGGTAGCACCTCCT	AACCTGGTTCATCATCGCTAA
Runx2	AAGTGCGGTGCAAACTTTCT	TCTCGGTGGCTGCTAGTGA
Col1A1	GCGAAGGCAACAGTCGCT	CTTGGTGGTTTTGTATTCGATGAC
Alpl	GTGCCAGAGAAAGAGAGAGA	TTTCAGGGCATTTTTCAAGGT
Bsp	CCCAGACAGCTGTCCTTCTGAA	ACGGTGCTGCTTTTTCTGATCG
Ocn	CTCACAGATGCCAAGCCCA	CCAAGGTAGCGCCGGAGTCT
Sost	AGCCTTCAGGAATGATGCCAC	TTTGGCGTCATAGGGATGGT
Opg	GTTCCTGCACAGCTTCACAA	AAACAGCCCAGTGACCATTC
Rankl	GCTCCGAGCTGGTGAAGAAA	CCCCAAAGTACGTCGCATCT
Mmp13	GCCCTGATGTTTCCCATCTA	TTTTGGGATGCTTAGGGTTG
c‐Fos	AGTTTATTTTGGCAGCCCACCG	AGGCAGACCTCCAGTCAAATCC

### Statistical analyses

For previous μCT analyses, an effect size of 1.43 on cortical thickness resulted in an estimated sample size of 9 animals per group. Using a conservative asymptotic relative efficiency value of 0.85, the sample size would need to be increased to 10 animals per group to attain a power of 0.8 at a two‐sided type I error rate of 0.05.

Data are presented as means ± SD and shown as individual values wherever possible. Outliers were measured by two different techniques and two different software packages because of the controversy of the threshold for outliers. First, we used the ROUT method in GraphPad, and then we confirmed it in SPSS using the 1.5 and 3 interquantile range rule multiplier (3IQR). Only if both the ROUT and the 3IQR were positive for an outlier was a value removed. Data were analyzed for normal distribution by Kolmogorov–Smirnov and Shapiro–Wilk tests and so‐called normal Q‐Q plot graphs. The equality of variance was determined with Levene's test. If normal distributions and/or equivalence of variances were not achieved, we used rank transformation and confirmed the normality by the previous tests. If both normal distribution and equality of variance were demonstrated, one‐way ANOVA was undertaken and results (*F* and *p* values) are provided in the panels. This was followed by a Tukey's multiple comparison test using SigmaStat software (IBM SPSS statistics, version 28, Sciences, Chicago, IL, USA) and GraphPad Prism 8 (2019 GraphPad Software, Inc., La Jolla, CA, USA). If we could not perform a parametric test, we used a nonparametric test such as the Kruskal–Wallis test. Overall Kruskal–Wallis *p* values are provided in the panels. For greater transparency, *p* values between 0.01 and 0.001 are provided in panels. We also note the statistical tests used for each panel in each figure legend. *p* < 0.05 was considered significant and shown in red in panels.

## Results

### Infusion of PTH (1–34) and abaloparatide caused a similar decrease in BMD in mice

To determine the catabolic effect of abaloparatide on bone, we continuously infused abaloparatide or PTH (1–34) or vehicle as controls to 2 month‐old wild type female mice for 2 weeks. In previous studies, we found that this age and sex produced the most consistent catabolic effect of infused PTH (1–34) in C57Bl/6J mice.^(^ ^36^ ^)^

**Fig. 1:**
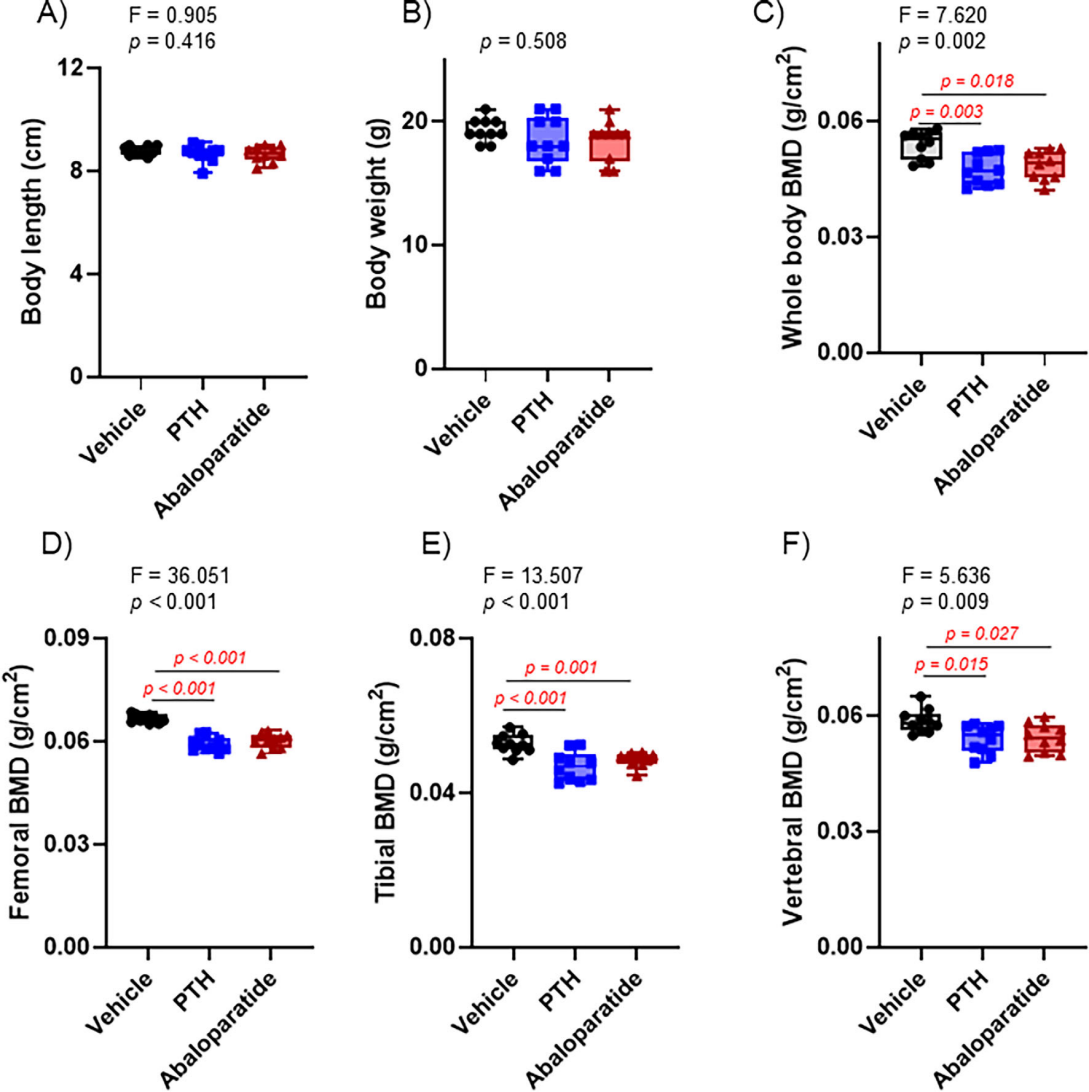
Infusion of PTH (1–34) and abaloparatide caused a similar decrease in BMD in mice. (*A*) Mouse body length at end of treatments. (*B*) Mouse body weight at end of treatments. At euthanasia, DEXA‐PIXImus was performed to measure BMD of (*C*) whole body, (*D*) femurs, (*E*) tibiae, and (*F*) lumbar vertebrae. Ten mice per group. Results are means ± SD. All data were analyzed for normality and equivalence of variance before a one‐way ANOVA followed by Tukey's multiple comparison test (*A*, *C*, *D*, *F*). The tibial BMD did not show an equivalence of variances, so a rank transformation was done before performing new normality and equivalence of variance tests and then a one‐way ANOVA followed by Tukey's multiple comparison test. The body weight analysis did not show equivalence of variance or a normal distribution, so we used Kruskal–Wallis, a nonparametric test. For greater transparency, ANOVA results (*F* and *p* values) or Kruskal–Wallis *p* values as well as individual *p* values (0.01 > *p* > 0.001) were provided in each panel. *p* < 0.05 was considered significant, and such values are shown in red.

All mice had the same range of body length and body weight at baseline as at the end of the treatments (Figure 1*A*,*B*). Compared with infused PBS, 2 weeks of infused abaloparatide or hPTH (1–34) caused a similar decrease in whole‐body BMD (Figure 1) and at several bone sites, such as femurs, tibiae, and lumbar vertebrae (Figure 1*D*–*F*). Abaloparatide and PTH (1–34) showed the same catabolic effect on bone. All these results were done in several experiments over time and from several litters of mice.

### Infused PTH (1–34) and abaloparatide had similar catabolic effects on cortical bone but differed in their effects on trabecular bone

After euthanasia, we fixed and stored femurs in 70% ethanol for μCT scanning to analyze and assess bone microarchitecture. The same femurs were sectioned after methacrylate embedding for histomorphometry studies to assess osteoblast and osteoclast activity.

**Fig. 2:**
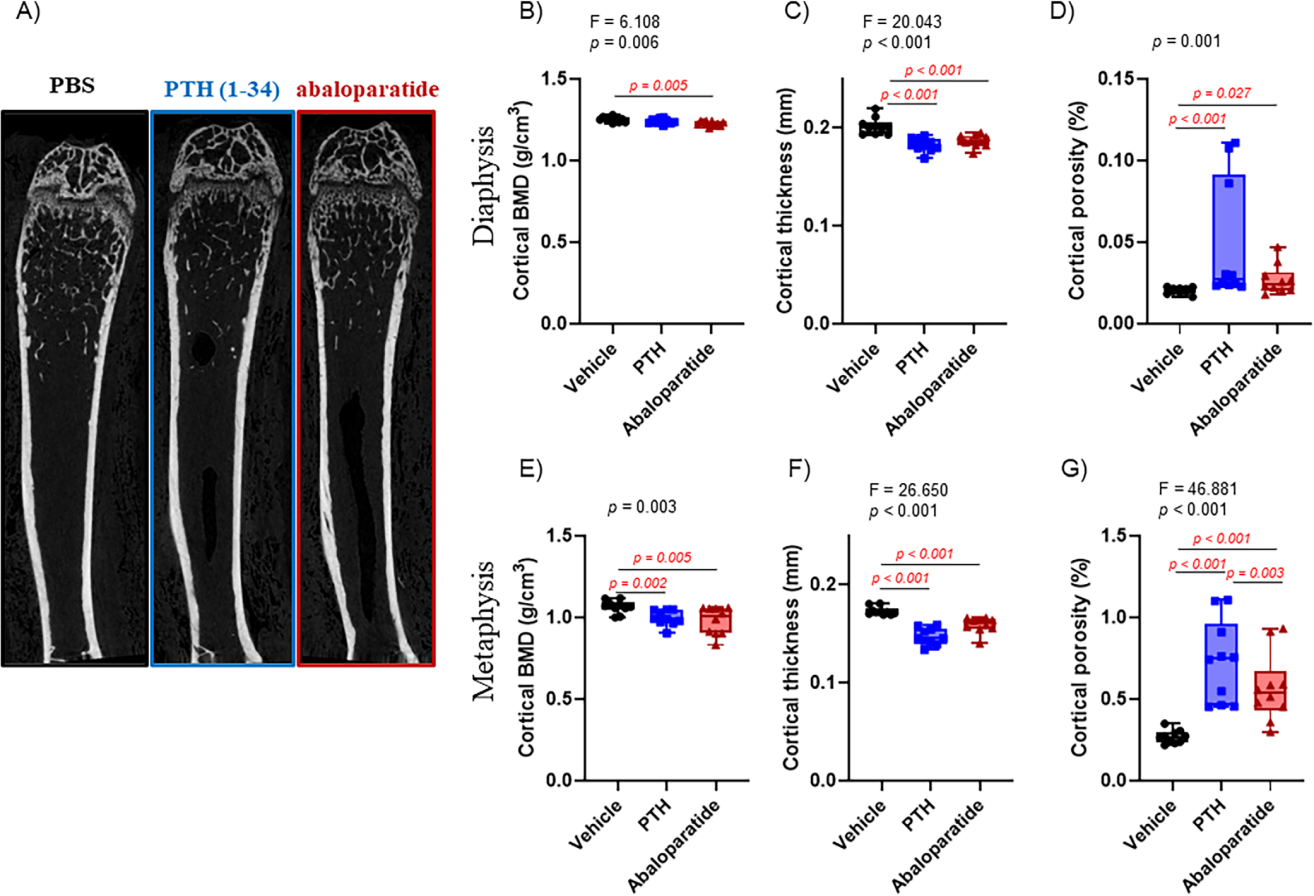
Infused PTH (1–34) and abaloparatide similarly caused bone loss in cortical bone. (*A*) Representative images of right femurs from female mice treated with infused PTH (1–34) or abaloparatide at 2 months of age for 2 weeks. Femurs were prepared for high‐resolution μCT and images were reconstructed with NRecon software. (*B*–*D*) A 600‐μm cortical volume corresponding to 62 slices of the mid‐diaphysis was examined for (B) cortical BMD, (*C*) cortical thickness, and (*D*) cortical porosity. (*E ‐ G*) A 970‐μm volume corresponding to 100 slices of the mid‐metaphysis was examined for cortical bone microarchitecture: (*E*) cortical BMD, (*F*) cortical thickness, (*G*) cortical porosity. Ten mice per group, results are means ± SD. All data were analyzed for normality and equivalence of variance before a one‐way ANOVA followed by Tukey's multiple comparison test (*B*, *C*). Some analyses did not show an equivalence of variances or normality, so a rank transformation was done before performing new normality and equivalence of variance tests and then a 1‐way ANOVA followed by Tukey's multiple comparison test (*F*, *G*). Others did not show equivalence of variance and/or a normal distribution, so we used Kruskal–Wallis, a nonparametric test (*D*, *E*). For greater transparency, ANOVA results (*F* and *p* values) or Kruskal–Wallis *p* values as well as individual *p* values (0.01 > *p* > 0.001) were provided in each panel. *p* < 0.05 was considered significant and is shown in red.

Similar to human hyperparathyroidism, both infused PTH (1–34) and abaloparatide had more effects on cortical bone than trabecular bone. With cortical bone, both treatments caused a significant decrease in cortical BMD and cortical thickness and an increase in cortical porosity, in the diaphysis and the metaphysis (Figure 2*A*–*G*). Only a few differences were observed between infused PTH (1–34) and abaloparatide: in the diaphysis, PTH (1–34) did not induce a significant decrease in BMD (Figure 2*B*) and in the metaphysis, PTH (1–34) showed a higher increase in cortical porosity compared with infused abaloparatide (Figure 2*G*).

**Fig. 3:**
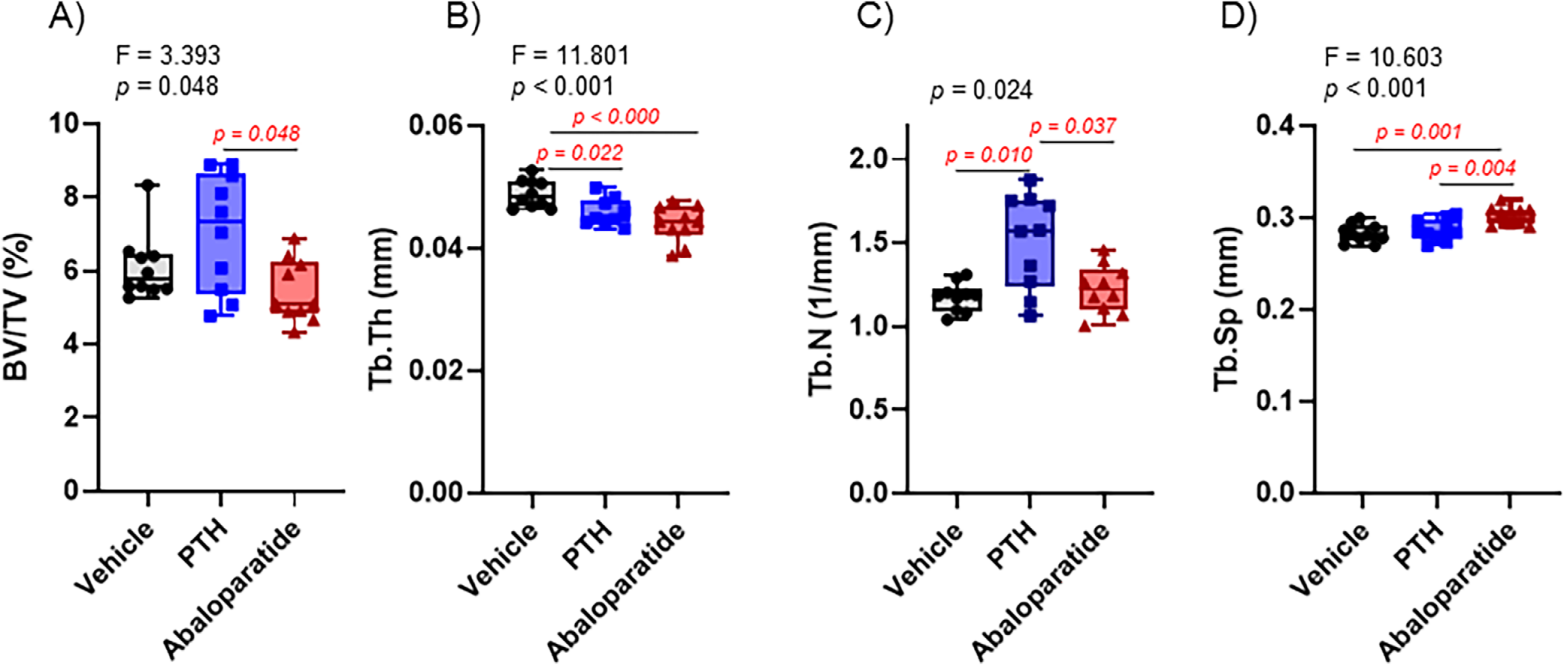
Infused PTH (1–34) and abaloparatide similarly decrease trabecular thickness without any change in bone volume. A 1940 μm volume corresponding to 200 slices of the mid‐metaphysis was examined for trabecular bone microarchitecture. This began 20 slices below the growth plate. (*A*) trabecular bone volume (BV/TV), (*B*) trabecular thickness (Tb.Th), (*C*) trabecular number (Tb.N), and (*D*) trabecular separation (Tb.Sp). Ten mice per group, results are means ± SD. All data were analyzed for normality and equivalence of variance before a one‐way ANOVA followed by Tukey's multiple comparison test (*B*, *D*). One analyses did not show an equivalence of variances or normality, so a rank transformation was done before performing new normality and equivalence of variance tests and then a one‐way ANOVA followed by Tukey's multiple comparison test (*A*). Another did not show equivalence of variance and/or a normal distribution, so we used Kruskal–Wallis, a nonparametric test (*C*). For greater transparency, ANOVA results (*F* and *p* values) or Kruskal–Wallis *p* values as well as individual *p* values (0.01 > *p* > 0.001) were provided in each panel. *p* < 0.05 was considered as significant, and such values are shown in red.

In contrast, but in accordance with some human and rodent studies, trabecular bone was not greatly affected by either of the infused peptide treatments. Whereas many published studies have shown a decreased trabecular bone volume in mice, in our mouse model and in our hands, infused PTH (1–34) has never significantly changed the trabecular bone volume.^(^ ^37^, ^38^ ^)^ No changes were observed in trabecular BMD (data not shown). Although a slight but not significant increase in the bone volume (BV/TV) was observed with PTH (1–34) treatment, infused abaloparatide presented a significantly lower BV/TV compared with infused PTH (1–34) that was slightly but insignificantly decreased compared with the control group (Figure 3*A*). Trabecular number showed the same pattern as the bone volume (Figure 3*C*): only infused PTH (1–34) induced a significant increase that was significantly greater than that seen with abaloparatide. Interestingly, both infused treatments caused a decrease in trabecular thickness (Figure 3*B*), whereas only abaloparatide caused an increase in trabecular separation (Figure 3*D*).

### Infused PTH (1–34) and abaloparatide both increased bone formation in cortical bone

**Fig. 4:**
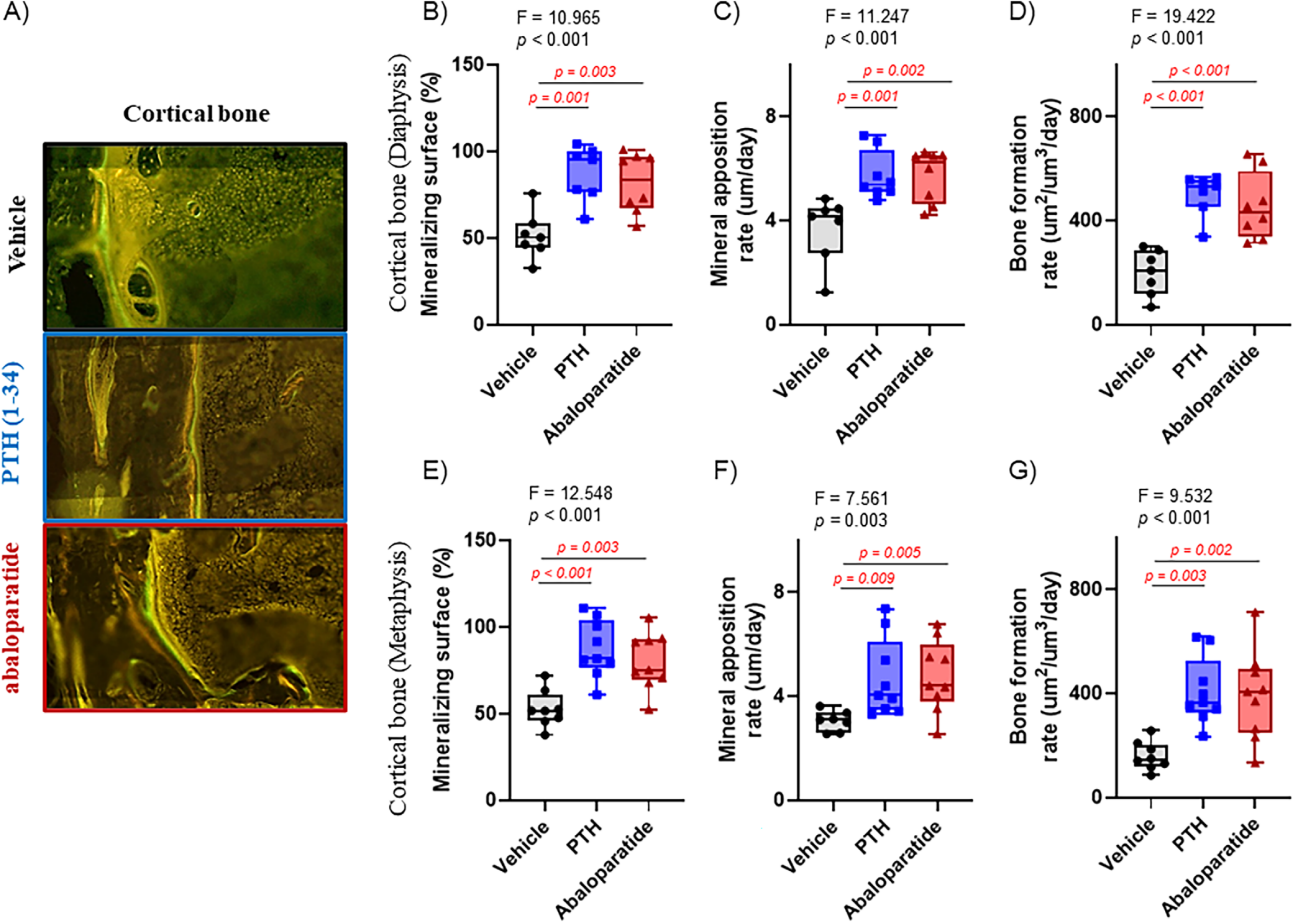
Infused PTH (1–34) and abaloparatide similarly stimulated cortical bone formation in mice. (*A*–*G*) Mice were injected with tetracycline at day 4 and with calcein at day 1 before death to determine bone formation. Femoral thin sections (10 μm) were obtained. (*A*) Representative images of right femurs showing differences in double labeling in cortical and trabecular bone in mice treated with infused PTH (1–34) or abaloparatide. Measurements were performed in the mid‐diaphysis for cortical dynamic analyses: (*B*) cortical mineralizing surface, (*C*) cortical mineral apposition rate, and (*D*) cortical bone formation rate but also in the metaphysis with (*E*) cortical mineralizing surface, (*F*) cortical mineral apposition rate, and (*G*) cortical bone formation rate. Seven to ten mice per group, results are means ± SD. Histomorphometry data were analyzed for normality and equivalence of variance before a one‐way ANOVA followed by a Tukey's multiple comparison test (*B*, *E*, *G*). Some analyses did not show an equivalence of variances or normality, so a rank transformation was done before performing new normality and equivalence of variance tests and then a one‐way ANOVA followed by Tukey's multiple comparison test (*C*, *D*, *F*). For greater transparency, ANOVA results (*F* and *p* values) or Kruskal–Wallis *p* values as well as individual *p* values (0.01 > *p* > 0.001) were provided in each panel. *p* < 0.05 was considered as significant, and such values are shown in red.

**Fig. 5:**
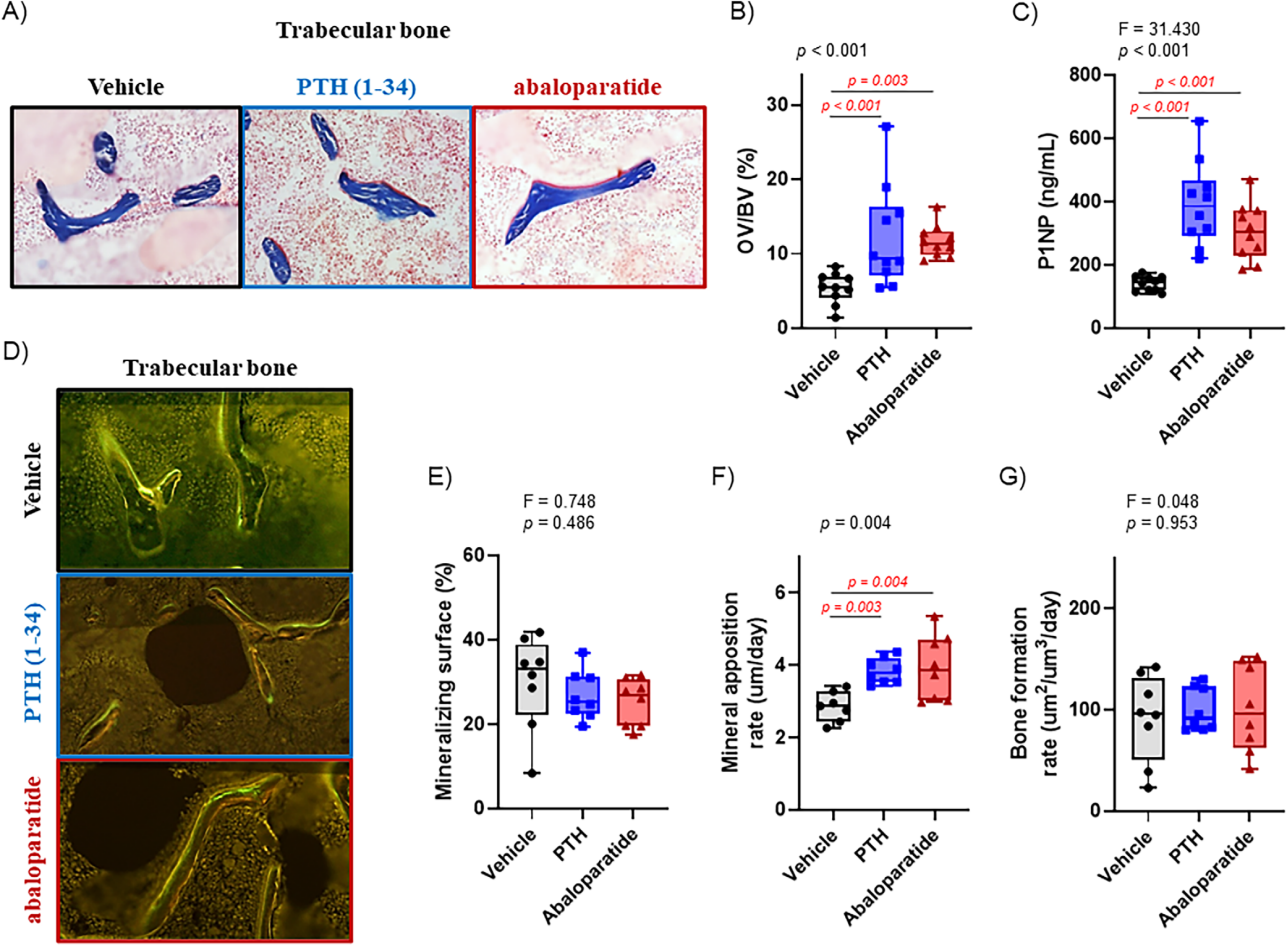
Infused PTH (1–34) and abaloparatide similarly stimulated trabecular osteoblast activity in mice. (*A*, *B*) Femoral thin sections (5 μm) were stained with Masson's trichrome. (*A*) Representative images of trabecular bone. Measurements were conducted in the secondary spongiosa for histomorphometry analysis of (*B*) osteoid volume (OV/BV). (*C*) At the end of the treatments, sera were collected. To analyze bone formation, serum P1NP levels were measured by ELISA. (*D*–*M*) Mice were injected with tetracycline at day 4 and with calcein at day 1 before death to determine bone formation. Femoral thin sections (10 μm) were obtained. (*D*) Representative images of right femurs showing differences in double labeling in trabecular bone in mice treated with infused PTH (1–34) or abaloparatide. Measurements were performed in the secondary spongiosa for trabecular dynamic analyses: (*E*) mineralizing surface, (*F*) mineral apposition rate, and (*G*) bone formation rate. Seven to ten mice per group, results are means ± SD. Histomorphometry data were analyzed for normality and equivalence of variance before a one‐way ANOVA followed by Tukey's multiple comparison test (*E*). Some analyses did not show an equivalence of variances or normality, so a rank transformation was done before performing new normality and equivalence of variance tests and then a one‐way ANOVA followed by Tukey's multiple comparison test (*C*, *G*). Others did not show equivalence of variance and/or a normal distribution so we used Kruskal–Wallis, a nonparametric test (*B*, *F*). For greater transparency, ANOVA results (*F* and *p* values) or Kruskal–Wallis *p* values as well as individual *p* values (0.01 > *p* > 0.001) were provided in each panel. *p* < 0.05 was considered significant, and such values are shown in red.

After 2 weeks of infused PTH (1–34) or abaloparatide, both treated groups had a similar increase in cortical bone formation, shown by double labeling (Figure 4*A*,*D*,*G*) for both peptides. With cortical bone, in the metaphysis and the diaphysis, both infused PTH (1–34) and abaloparatide generated a similar increase in active osteoblast number shown by mineralizing surface (Figure 4*B*,*E*), in osteoblast activity shown by the mineral apposition rate (Figure 4*C*,*F*), and, in consequence, bone formation rate (Figure 4*D*,*G*). This increase in bone formation caused an increase in the specific serum osteoblastic marker levels, P1NP (Figure 5*C*).

Although we observed a similar and significant decrease in trabecular thickness in both treated groups compared with the control group (Figure 3*B*), mice showed no changes in trabecular mineralizing surface and bone formation rate compared with the control group. Both PTH (1–34) and abaloparatide caused a significant increase in osteoid volume in the trabecular area showing increased osteoblast activity (Figure 5*A*,*B*). Only trabecular mineral apposition rate was slightly but significantly increased by infused PTH (1–34) and abaloparatide, which is consistent with the increased osteoid volume (Figure 5*D*–*G*).

### Infused PTH (1–34) and abaloparatide differentially regulate osteoblastic gene expression

**Fig. 6:**
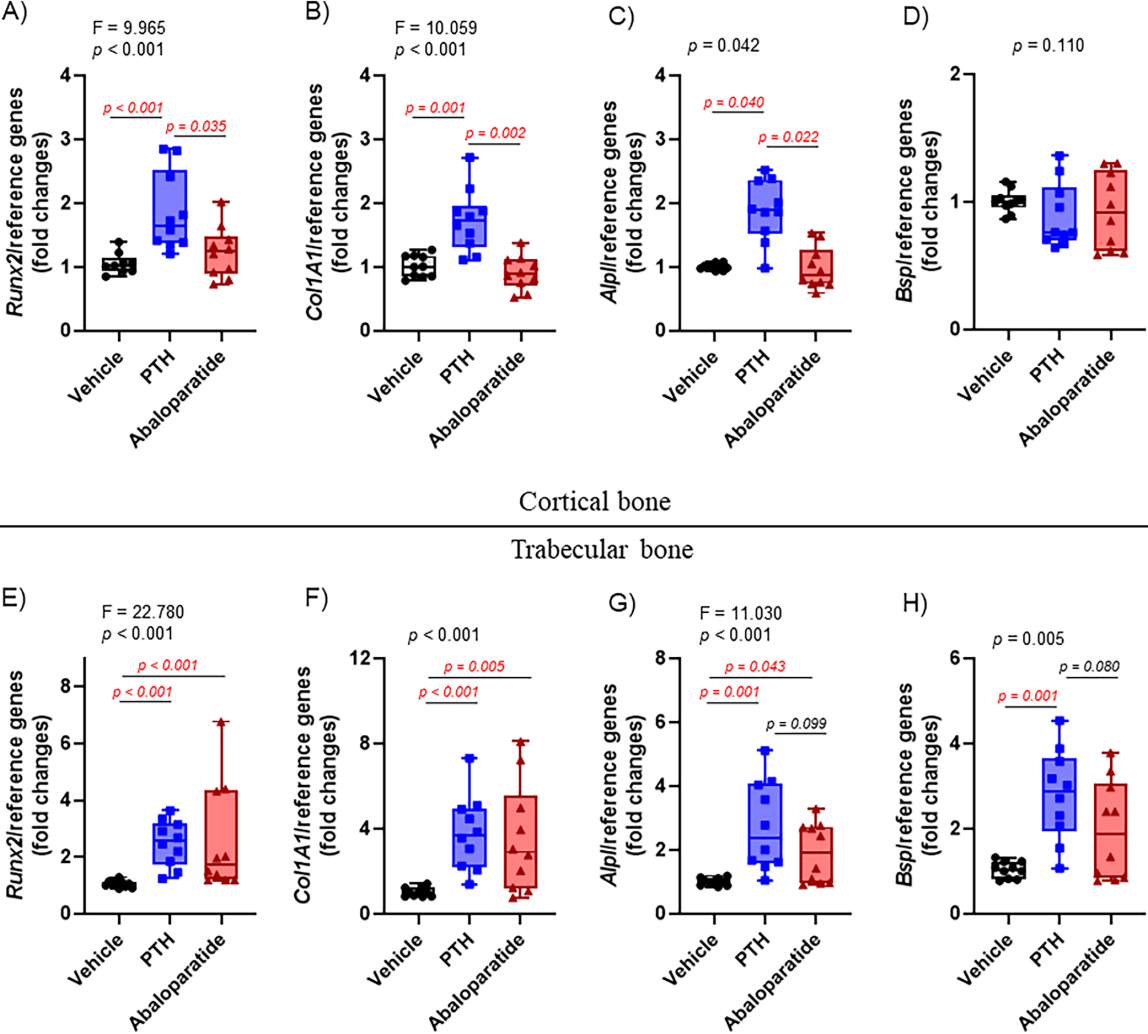
PTH (1–34) and abaloparatide differentially regulated osteoblastic gene expression. Two tibiae from each animal were divided into subcortical trabecular‐rich bone and cortical bone (osteocyte‐rich bone). Total RNA was isolated, and qRT‐PCR was performed. (*A*–*D*) Osteoblastic gene expression was measured in the osteocyte‐rich cortical tibial bone: (*A*) *Runx2*, (*B*) *Col1A1*, (*C*) *Alpl*, and (*D*) bone sialoprotein or *Bsp*. (*E*–*H*) Osteoblastic gene expression was measured in the trabecular‐rich tibial bone: (*E*) *Runx2*, (*F*) *Col1A1*, (*G*) *Alpl*, and (*H*) *Bsp*. Expression of all genes was compared with housekeeping genes and shown as fold change compared with control animals. Ten mice per group, results are means ± SD. These data were analyzed for normality and equivalence of variance before a one‐way ANOVA followed by Tukey's multiple comparison test (*A*, *B*). Some analyses did not show an equivalence of variances or normality, so a rank transformation was done before performing new normality and equivalence of variance tests and then a one‐way ANOVA followed by Tukey's multiple comparison test (*E*, *G*). Most did not show equivalence of variance and/or a normal distribution, even after a rank transformation, so we used Kruskal–Wallis, a nonparametric test (*C*, *D*, *F*, *H*). For greater transparency, ANOVA results (*F* and *p* values) or Kruskal–Wallis *p* values as well as individual *p* values (0.01 > *p* > 0.001) were provided in each panel. *p* < 0.05 was considered as significant, and such values are shown in red.

Because of a high bone turnover phenotype in hyperparathyroidism, we expected to see an increase in bone formation (shown in Figures 4 and 5) caused by more active osteoblasts. To confirm the anabolic action of both infused peptides, we measured osteoblastic gene expression (Figure 6*A*–*H*). In previous studies, infused or intermittent PTH was shown to enhance many genes involved in bone formation, such as the osteoblast‐specific runt‐related transcription factor 2 (*Runx2*), alkaline phosphatase (*Alpl*), collagen type I alpha 1 (*Col1A1*), and osteocalcin (*Ocn*).^(^ ^39^, ^40^ ^)^ Globally, we observed significant differences with infused abaloparatide on these osteoblastic genes compared with PTH (1–34). This demonstrated that even if both infused peptides had the same outcome and action on osteoblast number and activity, they did not act in the same way through their common receptor and, as a consequence, on osteoblastic gene expression (Table 1).

**Fig. 7:**
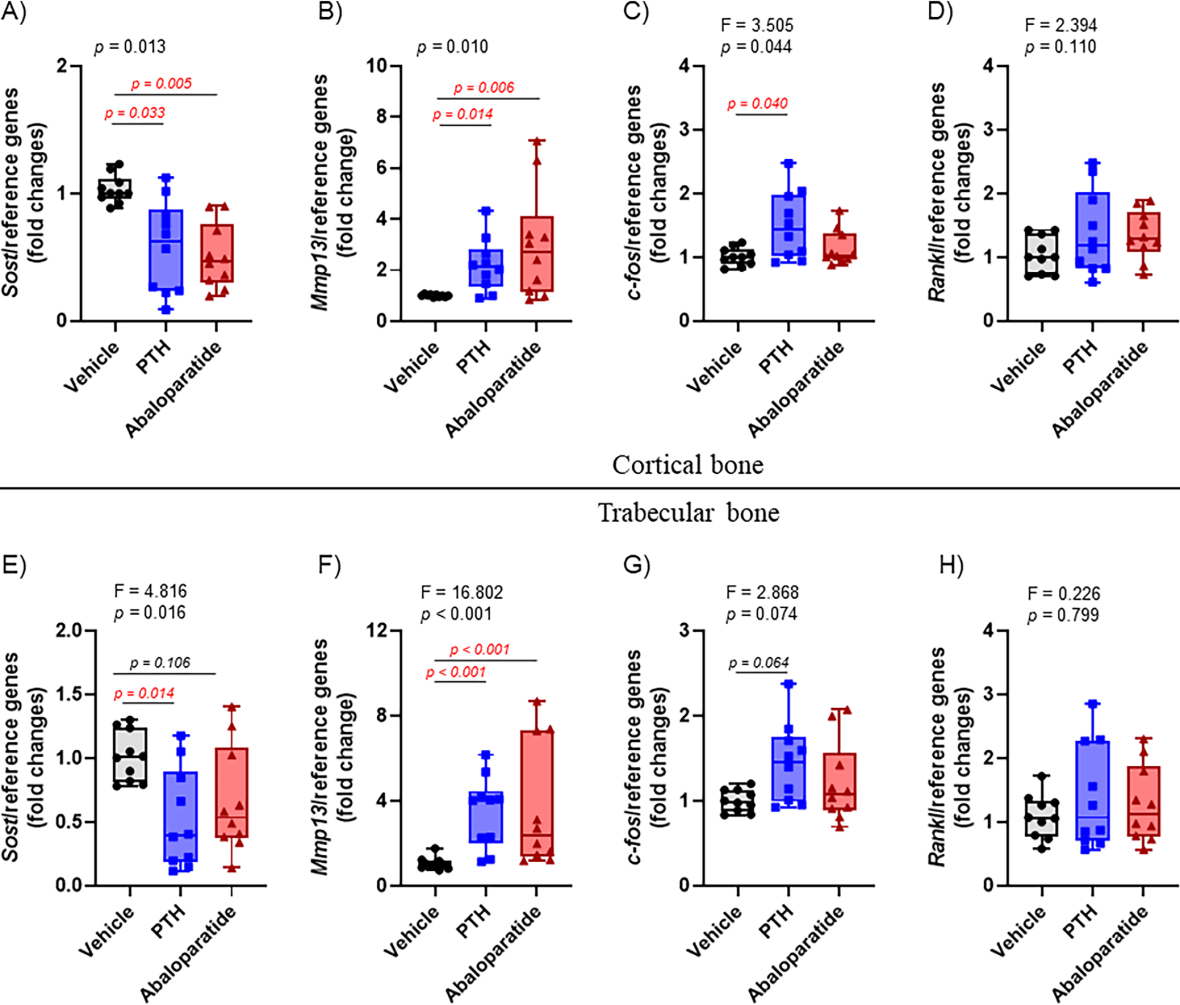
PTH (1–34) and abaloparatide differentially regulated PTHR1‐responsive osteoblastic gene expression. Two tibiae from each animal were divided into subcortical trabecular‐rich bone and cortical bone (osteocyte‐rich bone). Total RNA was isolated, and qRT‐PCR was performed. (*A*–*D*) PTHR1‐responsive gene expression was measured in the osteocyte‐rich cortical tibial bone: (*A*) *Sost*, (*B*) *Mmp13*, (*C*) *c‐fos*, and (*D*) *Rankl*. (*E*–*H*) PTHR1‐responsive gene expression was measured in the trabecular‐rich tibial bone: (*E*) *Sost*, (*F*) *Mmp13*, (*G*) *c‐fos*, and (H) *Rankl*. Expression of all genes was compared with housekeeping genes and shown as fold change compared with control animals. Ten mice per group, results are means ± SD. These data were analyzed for normality and equivalence of variance before a one‐way ANOVA followed by Tukey's multiple comparison test (*D*). Some analyses did not show an equivalence of variances or normality, so a rank transformation was done before performing new normality and equivalence of variance tests and then a one‐way ANOVA followed by Tukey's multiple comparison test (*C*, *E*, *F*, *G*, *H*). Some did not show equivalence of variance and/or a normal distribution, even after a rank transformation, so we used Kruskal–Wallis, a nonparametric test (*A*, *B*). For greater transparency, ANOVA results (*F* and *p* values) or Kruskal–Wallis *p* values as well as individual *p* values (0.01 > *p* > 0.001) were provided in each panel. *p* < 0.05 was considered significant, and such values are shown in red.

In cortical bone, the infused PTH (1–34) had quite different effects from infused abaloparatide. Infusion of abaloparatide did not affect the expression of *Runx2*, *Col1A1*, *Alpl*, or *Bsp* (Figure 6*A*–*C*), whereas, as expected, infused PTH (1–34) caused an increase in the osteoblastic gene expression of *Runx2*, *Col1A1*, and *Alpl* (Figure 6*A*–*C*). No changes were observed in *Bsp* expression (Figure 6*D*). Interestingly, both infused treatments reduced *Sost* expression, a well‐known PTH‐responsive gene (Figure 7*A*).

In contrast to the cortical/osteocyte‐rich bone, both PTH (1–34) and abaloparatide increased *Runx2*, *Col1A1*, and *Alpl* expression (Figure 6*E*–*G*) in trabecular‐rich bone. But only infused PTH (1–34) significantly increased *Bsp* and decreased *Sost* expression compared with infused abaloparatide and control groups (Figures 6*H* and 7*E*). For these latter genes, infused abaloparatide seemed to have the same tendency compared with PTH (1–34), but to a lesser and insignificant degree compared with the control group.

### Infusion of both PTH (1–34) and abaloparatide increased bone resorption

**Fig. 8:**
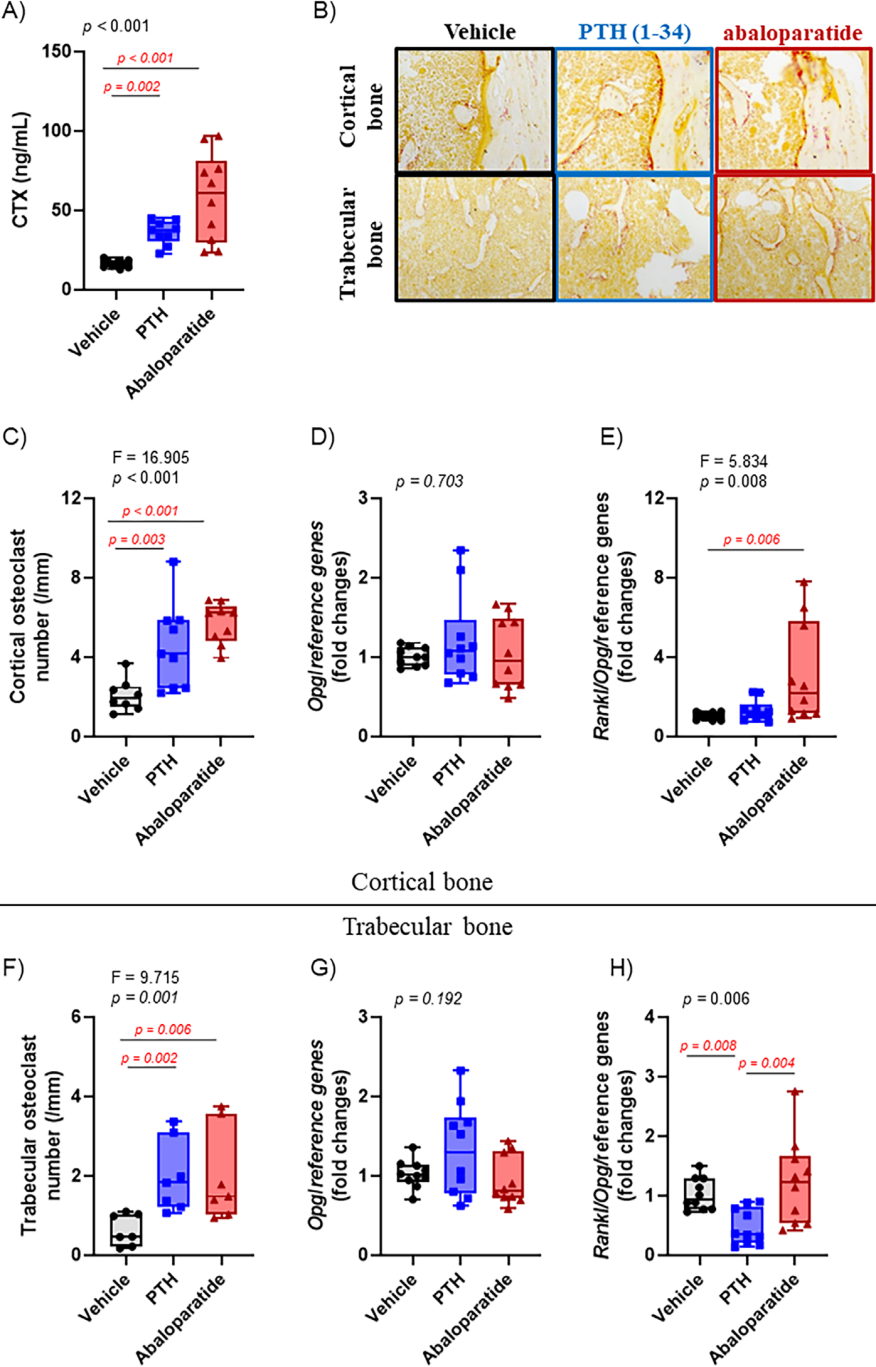
Both PTH (1–34) and abaloparatide increased bone resorption in mice. To assess bone resorption, (*A*) serum CTX levels were measured by ELISA in female mice infused with PTH (1–34) or abaloparatide. (*B*, *C*, *F*) Femurs were fixed in 70% ethanol, dehydrated, and embedded in methacrylate. TRACP staining was done in order to count osteoclasts on bone surfaces, (*C*) in cortical bone in the mid‐diaphysis, and (*F*) in trabecular area. (*B*) Representative images of TRACP staining in the cortical and trabecular bones. Two tibiae from each animal were divided into subcortical trabecular‐rich bone and cortical bone (osteocyte‐rich bone). Total RNA was isolated, and qRT‐PCR was performed. Catabolic gene expression was measured in the cortical bone for (*D*) *Opg*, (*E*) *Rankl*/*Opg* ratio, and in the trabecular‐rich fraction for (*G*) *Opg* and (*H*) *Rankl*/*Opg* ratio. Expression of all genes was compared with housekeeping genes and shown as fold change compared with control animals. Seven to ten mice per group, results are means ± SD. All data were analyzed for normality and equivalence of variance. Most analyses did not show an equivalence of variances or normality, so a rank transformation was done before performing new normality and equivalence of variance tests and then a one‐way ANOVA followed by Tukey's multiple comparison test (*C*, *E*, *F*). Others did not show equivalence of variance and/or a normal distribution, even after a rank transformation, so we used Kruskal–Wallis, a nonparametric test (*A*, *D*, *G*, *H*). For greater transparency, ANOVA results (*F* and *p* values) or Kruskal–Wallis *p* values as well as individual *p* values (0.01 > *p* > 0.001) were provided in each panel. *p* < 0.05 was considered significant, and such values are shown in red.

Serum analyses showed an increase in serum CTX levels that indicate an increase in bone resorption with both infused PTH (1–34) and abaloparatide compared with the control group (Figure 8*A*). The increase in bone resorption was confirmed by histomorphometry, which showed a similar doubling of osteoclast numbers in both trabecular and cortical bone (Figure 8*B*,*C*,*F*) with both of the infused peptides. The increased CTX appeared higher with infused abaloparatide compared with infused PTH (1–34), but in an insignificant manner. A similar trend was observed with the cortical osteoclast number.

The serum calcium levels were unchanged, whereas serum phosphate levels were significantly lower after PTH (1–34) infusion and not significantly lower after abaloparatide infusion.

In hyperparathyroidism, the receptor activator of the nuclear factor kappa B ligand (RANKL) was shown to be hyperexpressed and, as a consequence, to be responsible for the catabolic effect of PTH (1–34) in bone.^(^ ^41^, ^42^ ^)^ RANKL is involved in the major pathways utilized in PTH‐induced resorption: the *Rankl*/*Rank*/*Opg* system. The balance between RANKL and osteoprotegerin (OPG) is responsible for inducing osteoclastogenesis. Previous studies showed that PTH (1–34) acts on osteoclastogenesis by increasing *Rankl* expression^(^ ^43^ ^)^ and decreasing *Opg* expression.^(^ ^44^ ^)^ In our mouse model, in cortical and trabecular area, no significant changes were observed in *Rankl* (Figure 7*D*,*H*) and *Opg* expression (Figure 8*D*,*H*) for either treatment compared with the control group. Nevertheless, because of a slight and insignificant increase in *Rankl* and decrease in *Opg* expression, infused abaloparatide significantly increased the ratio of *Rankl/Opg*, but only in cortical bone (Figure 8*E*,*H*). In contrast, in trabecular bone, because of a slight and insignificant increase in *Opg*, infused PTH (1–34) significantly decreased the ratio of *Rankl/Opg*, whereas infused abaloparatide had no effect (Figure 8*H*).

Another protein, regulated by PTH (1–34), could be involved in this increased bone resorption. PTH (1–34) has been shown to modulate the complex secondary response in the expression of matrix metalloproteinase 13 (*Mmp13*), partially responsible for extracellular matrix degradation.^(^ ^45^, ^46^ ^)^ Here, both infused PTH (1–34) and abaloparatide elicited the same increase in *Mmp13* expression and in both trabecular and cortical bone (Figure 7*B*,*F*).

c‐Fos is an immediate early downstream PTH effector in osteoblasts by way of PTHR1/cAMP/PKA signaling. It has been shown that PTH (1–34) induces a quicker and higher *c‐Fos* mRNA expression compared with abaloparatide.^(^ ^21^, ^47^ ^)^ In our mouse model, only infused PTH (1–34) caused a slight but significant increase in *c‐Fos* expression in cortical bone (Figure 7*C*). No significant changes were observed in trabecular bone (Figure 7*G*).

## Discussion

In our previous in vivo study, we showed that abaloparatide, at the same dose, had the same anabolic effects on bone as PTH (1–34).^(^ ^30^ ^)^ With these intermittent peptide treatments, both peptides, at the same dose, similarly increase bone formation and, as we showed for the first time, bone resorption. Likewise, and in order to compare their specific catabolic effects on bone, we conducted a head‐to‐head comparison of both peptides by infusing PTH (1–34) and abaloparatide, at the same dose, similar peptide content, and time point, in wild‐type mice. Sustained high PTH serum levels or continuous infusion of PTH are well known to cause a catabolic effect associated with a decreased bone mass due to a higher increase in bone resorption than bone formation.^(^ ^41^ ^)^ In humans, abaloparatide is used therapeutically at 80 μg/day compared with teriparatide at 20 μg/day to obtain equivalent anabolic effects in humans after 24 weeks of daily injections in total hip and femoral neck BMD.^(^ ^26^ ^)^ However, the increase in bone formation markers was dose‐dependent, but similar increases in a bone resorption marker were observed, and all were lower compared with teriparatide.^(^ ^26^, ^48^ ^)^ It is difficult to do a real comparison when the dosages are not the same. Several publications have compared the anabolic effects of abaloparatide with PTH (1–34) but with a range of dosages, mouse age/sex, and duration of treatments.^(^ ^49^ ^)^ In all cases, when PTH (1–34) was used at the same dose as abaloparatide, the effects were equal.^(^ ^49^ ^)^ In mouse models, the standard dose of PTH (1–34) for anabolic treatment is 80 μg/kg BW injected daily in order to have the same effects observed in humans. We used a common mouse model and continuously infused 2‐month‐old female mice with the same dose of PTH (1–34) and abaloparatide as the anabolic study of PTH (1–34).^(^ ^32^ ^)^ In our laboratory, we have already tried infusing several PTH dosages (40 and 80μg/kg/day), at several ages (8 weeks, 4 or 6 months‐old), both sexes, and for 2 or 4 weeks of infusion. We have observed over the years and experiments that infusion in 8‐week‐old female mice at 80 μg/kg BW/day for 2 weeks are the optimal conditions.^(^ ^37^, ^38^ ^)^ In this way, we were able to investigate the comparative catabolic effects of both peptides.

Some authors have also used fourfold higher doses of each of these. It is evident that a higher dose of PTH (1–34) yields a greater effect than its lower dose,^(^ ^50^ ^)^ whereas in all cases, PTH (1–34), at the same dose as abaloparatide, has the same effect.^(^ ^49^ ^)^ We expect that the use of abaloparatide at a fourfold higher dosage (320 μg/kg/day) would cause more bone resorption or more side effects. In fact, Arlt et al.^(^ ^51^ ^)^ found a significantly greater serum RANKL/OPG ratio with four times higher abaloparatide dose. Indeed, it has already been shown that in humans, PTHrP (1–36) needs to be used at a dose 20 times higher than PTH to have the same anabolic effects, but at this high dose, PTHrP (1–36) causes hypercalcemia.^(^ ^52^ ^)^

Previous studies demonstrated that, in vitro, abaloparatide had a less stimulatory effect on cAMP/PKA/CRTC (cAMP‐regulated transcriptional coactivator) signaling than PTH (1–34), which results in lower cAMP levels, less PKA activation, and some differences in gene expression.^(^ ^20^, ^21^ ^)^ Nevertheless, in our previous in vivo study, both intermittent PTH (1–34) and abaloparatide similarly stimulated bone formation and bone resorption, which led to the same increased bone volume and osteoanabolic actions. In our continuous infusion mouse model, at the same dose, we confirmed that both peptides had the same catabolic effects on bone formation and resorption with the same high bone turnover. In fact, we found that PTH (1–34) and abaloparatide caused very similar decreases in BMD and cortical bone thickness, increased cortical bone formation rate, and, importantly, increased osteoclast numbers. They both stimulated the production of P1NP and CTX, confirming a situation of high bone turnover. As in other rodent studies and human hyperparathyroidism, infusion of PTH and abaloparatide led to a decrease in cortical thickness and an abnormal remodeling of trabecular bone.^(^ ^38^, ^53^, ^54^, ^55^, ^56^ ^)^ With both treatments, trabecular bone in this catabolic mouse model shows no major changes in bone volume but an increase in trabecular separation (which reflects increased osteoclast activity) and decreased trabecular thickness. The latter indicates a decrease in osteoblast number and activity, but increased bone formation is observed. This could be explained by the increase in osteoid volume and appearance of bone marrow fibrosis (data not shown), called osteitis fibrosa.^(^ ^55^, ^57^ ^)^ This is not uncommon because it is also observed in 50% of human biopsies in hyperparathyroidism.^(^ ^55^ ^)^ The faster process of bone resorption could also blunt bone formation, which takes longer to occur.

Previously, we showed that abaloparatide is a weaker activator of cAMP/PKA/CREB signaling compared with PTH (1–34), and it is associated with a lower stimulation of *c‐Fos* and *Rankl* expression in mouse osteoblasts in vitro and in vivo.^(^ ^21^ ^)^ With infusion, *c‐Fos* expression is only increased by PTH (1–34) in cortical bone. The lack of abaloparatide effect on *c‐Fos* gene expression could be explained by a smaller and shorter stimulation compared with PTH (1–34).^(^ ^21^, ^30^ ^)^ Similar to intermittent treatment, no changes in *c‐Fos* expression were observed in trabecular bone.^(^ ^30^ ^)^

The regulation of *Rankl* and *Opg* gene expression seems to be more complicated. It has been shown that continuous PTH induces bone resorption by activating osteoclast differentiation indirectly through increased *Rankl* and decreased *Opg* (RANKL receptor decoy) expression by osteoblasts.^(^ ^5^, ^58^ ^)^ In our previous study, both intermittent PTH (1–34) and abaloparatide increased *Rankl/Opg* only in trabecular bone and only 18 h after the last peptide injection. Abaloparatide had a more rapid effect, with an elevated ratio 4 h after the last injection.^(^ ^30^ ^)^ In both mouse models, *Opg* expression was not changed by any treatments, in either bone area. Unexpectedly, we observed no significant changes in *Rankl* expression with infused PTH (1–34) or abaloparatide, in either cortical or trabecular bones. This could be explained by the fact that comparing intermittent and continuous treatments, smaller effects on gene expression are observed with continuous treatment. In rats, *Rankl* is dramatically but transiently increased by intermittent PTH (1–34), whereas this upregulation is moderate but sustained by continuous PTH treatment.^(^ ^11^ ^)^ However, these data were obtained in rats, which are known to be more susceptible to PTH (1–34) treatment compared with mice. In our catabolic mouse model, abaloparatide seemed to have a significant effect on *Rankl/Opg* caused by slightly but insignificantly decreased *Opg* and increased *Rankl* expression, in cortical bone. In trabecular bone, abaloparatide had no effect on *Rankl/Opg*. It is also possible that there is a greater increase in *Rankl/Opg* at an earlier time point in the 14 days of infusion. In addition, although abaloparatide seems to have an equipotent effect on PTHR1/SIK/HDAC4 signaling compared with PTH (1–34),^(^ ^21^ ^)^ abaloparatide has a weaker stimulation of the PTHR1/SIK/CRTC pathway, which is responsible for *Rankl* transcription. This latter pathway appears to be less sensitive to cAMP/PKA activity^(^ ^21^ ^)^ and, as a consequence, less active in vitro with abaloparatide compared with PTH (1–34), which produces a prolonged production of cAMP compared with abaloparatide.^(^ ^21^ ^)^ The pharmacokinetic profile of PTHR1‐responsive genes could be another important factor in this paradox between gene expression and physiological effect.

In hyperparathyroidism, monocyte chemoattractant protein 1 (MCP‐1, CCL2) levels are increased, as are those of RANKL. In addition, MCP‐1 fell following parathyroidectomy in humans.^(^ ^59^, ^60^, ^61^ ^)^ The chemokine is highly upregulated by daily hPTH (1–34) injections and moderately by continuous infusion of PTH in rats.^(^ ^11^, ^62^ ^)^ MCP‐1 is also necessary for both PTH's anabolic and catabolic effects, which were abolished in MCP‐1^−/−^ mice.^(^ ^33^, ^63^ ^)^ In a previous study, we showed that the constant elevated levels of MCP‐1, caused by continuous PTH, recruits and differentiates monocytes, macrophages, and osteoclasts.^(^ ^38^ ^)^ The constant elevated levels of MCP‐1 may enhance osteoclastic bone resorption and could be an alternative for osteoclastogenesis induced by changes in *Rankl/Opg*. It has also been shown that MCP‐1 stimulates osteoclast formation inducing bone loss in prostate cancer bone metastasis.^(^ ^64^, ^65^ ^)^ Another gene that could be regulated by the infusion of both peptides is *Csf1*. PTH stimulates *Csf1* expression in osteoblasts, which then binds its receptor c‐FMS, which is highly expressed on osteoclasts at each stage of maturation from precursors to mature osteoclasts.^(^ ^66^, ^67^ ^)^ Both CSF1 and MCP‐1 would also regulate macrophages, and we have observed that PTH (1–34) increases the number of macrophages in bone marrow.^(^ ^63^ ^)^ These cells, as well as osteoclast precursors, could contribute to the increased catabolic activity. PTH infusion in rodents also increases serum levels of IL‐6, which is an important mediator of bone resorption, and this cytokine could also be implicated here.^(^ ^68^ ^)^

In summary, abaloparatide has the same catabolic effects as PTH when continuously infused in intact female mice. Both peptides showed the same effects on bone formation and bone resorption in cortical and trabecular bones. Nonetheless, even if infused abaloparatide has the same physiological effects as PTH (1–34), it differs in the regulation of some genes, suggesting different cellular and molecular effects. More studies are needed to better understand the PTH or abaloparatide/PTHR1 pathway and to develop better anabolic treatments for osteoporosis.

## Author Contributions

**Carole Anne Le Henaff:** Conceptualization; data curation; formal analysis; investigation; methodology; supervision; validation; visualization; writing – original draft; writing – review and editing. **Brandon Finnie:** Formal analysis; methodology. **Maria Pacheco:** Formal analysis; methodology. **Zhiming He:** Formal analysis; methodology. **Joshua Hayes Johnson:** Formal analysis; methodology.

## Funding Information

This work was supported by NIH Grants R01 DK047420 and S10 0D010751 (to NCP)

### Peer Review

The peer review history for this article is available at https://publons.com/publon/10.1002/jbm4.10710.

## Data Availability

The data that support the findings of this study are available from the corresponding authors upon reasonable request.
